# Gas emission during laparoscopic colorectal surgery using a bipolar vessel sealing device: A pilot study on four patients

**DOI:** 10.1186/1754-9493-2-22

**Published:** 2008-09-19

**Authors:** Martin Hübner, Markus W Sigrist, Nicolas Demartines, Michele Gianella, Pierre A Clavien, Dieter Hahnloser

**Affiliations:** 1 Department of Visceral and Transplantation Surgery, University Hospital Zürich, Switzerland; 2 Institute for Quantum Electronics, ETH Zürich, Switzerland; 3 Department of Visceral Surgery, CHUV, Lausanne, Switzerland

## Abstract

**Background:**

Dissection during laparoscopic surgery produces smoke containing potentially toxic substances. The aim of the present study was to analyze smoke samples produced during laparoscopic colon surgery using a bipolar vessel sealing device (LigaSure™).

**Methods:**

Four consecutive patients undergoing left-sided colectomy were enrolled in this pilot study. Smoke was produced by the use of LigaSure™. Samples (5,5l) were evacuated from the pneumoperitoneum in a closed system into a reservoir. Analysis was performed with CO_2_-laser-based photoacoustic spectroscopy and confirmed by a Fourier-transform infrared spectrum. The detected spectra were compared to the available spectra of known toxins.

**Results:**

Samples from four laparoscopic sigmoid resections were analyzed. No relevant differences were noted regarding patient and operation characteristics. The gas samples were stable over time proven by congruent control measurements as late as 24 h after sampling. The absorption spectra differed considerably between the patients. One broad absorption line at 100 ppm indicating H_2_O and several unknown molecules were detected. With a sensitivity of alpha min ca 10^-5 ^cm^-1 ^no known toxic substances like phenol or indole were identified.

**Conclusion:**

The use of a vessel sealing device during laparoscopic surgery does not produce known toxic substances in relevant quantity. Further studies are needed to identify unknown molecules and to analyze gas emission under various conditions.

## Background

Bipolar vessel sealing devices (LigaSure™) are frequently used in laparoscopic surgery for secure hemostasis, fast dissection and limited collateral tissue damage [1,2]. Smoke is produced by the combustion of organic tissue [3-5]. The main components of the smoke are gaseous compounds, bio-aerosols, volatile organic compounds, cellular material and even viruses [3,4,6,7].

During laparoscopic surgery a specific danger arises for the patient because potentially toxic substances are generated in high concentrations (closed system) in the abdominal cavity and can be absorbed by the peritoneum [6,8,9]. Potential hazards include CO toxicity, cytotoxicity and port site metastases [4,8,9]. The composition of surgical smoke produced by laparoscopic surgery is likely to be different compared to open surgery, as procedures are performed in a CO_2 _atmosphere [4,6].

Little is known about the generation of surgical by-products during laparoscopy. The aim of this study was to analyze gas samples produced by use of LigaSure™ for toxic substances during laparoscopic colorectal surgery.

## Methods

### Patients

Patients were recruited from a prospective randomized trial comparing three different devices in laparoscopic colon surgery. Samples for the presented pilot study were obtained from four consecutive patients randomized to be operated by use of LigaSure™. Data acquisition for the randomized trial and the pilot study did not interfere. The procedure was approved by the institutional ethic committee (University of Zürich), and an informed consent was obtained for each patient. The study was registered at clinicaltrials.gov and was allocated the number NCT00517608.

### Experimental details

**Figure 1:**
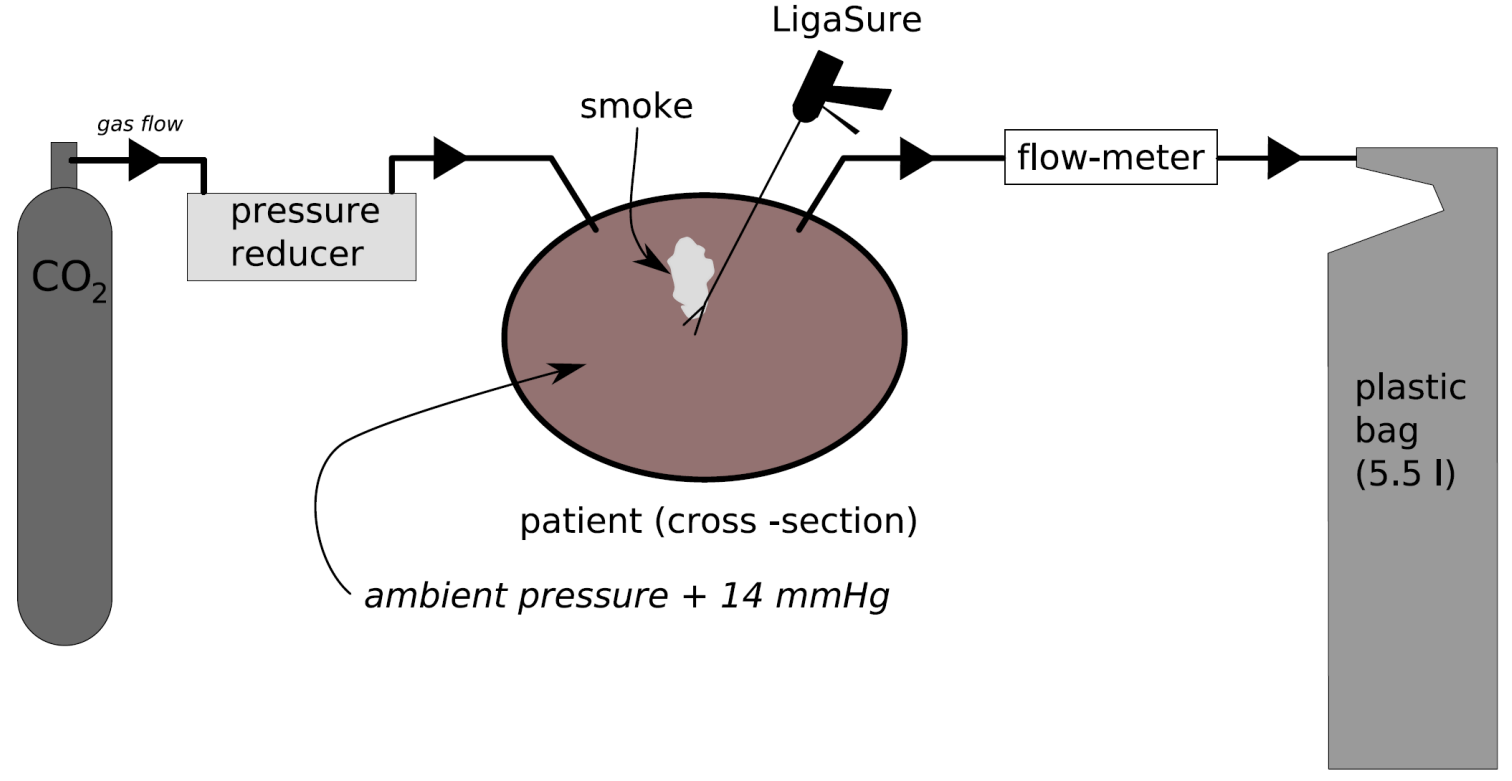
**Experimental set-up for the gas sampling during laparoscopic surgery**. Laparoscopic colectomy was performed in a CO_2 _atmosphere at 14 mmHg overpressure using LigaSure™ as dissection device. Smoke samples were evacuated from the pneumoperitoneum via one trocar and collected into Teflon bags.

Pneumoperitoneum was created with carbon dioxide at 14 mmHg overpressure in a closed system. Smoke samples (5,5l) were evacuated during laparoscopic dissection with LigaSure™ (Valleylab, Boulder, CO, USA) through one trocar and collected in a closed system into Teflon bags (Linde, Plastigas) as depicted in Fig 1. One bag filled directly with CO_2 _from the bottle served as control. The gas samples were analyzed using a ^13^CO_2 _laser photoacoustic (PA) spectrometer between 10.7 and 11.3 μm [5,10]. Measurements were performed approximately 5 hours after sampling and lasted 2 hours. One sample was additionally analyzed with a Fourier transform infrared spectrometer (FTIR) (Bomem, Model DA8) to confirm laser PA measurements.

## Results

Between 26^th ^of January and 2^nd ^March 2006, four patients undergoing laparoscopic left-sided colectomy were randomized to be operated by the use of LigaSure™. These four consecutive patients were similar regarding relevant patient characteristics and no particular events were noted during any of these operations (e.g. prolonged operation time, conversion to laparotomy, intestinal perforation, heavy bleeding).

**Figure 2:**
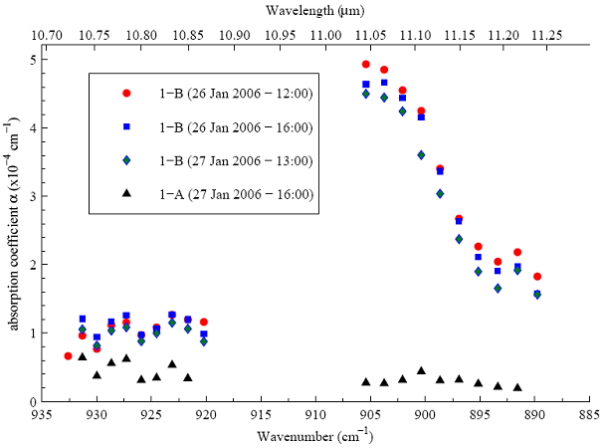
**Stability of the smoke sample over time and comparison to a control sample**. Photoacoustic absorption spectra of smoke and control sample taken on the 26^th ^of January. The smoke sample (1-B) was measured at three different times and compared to a control sample (1-A) which was directly retrieved from the carbon dioxide bottle.

The first sample (26^th ^of January) was analyzed at three different times and compared to a control sample which was retrieved directly from the carbon dioxide bottle (Fig 2). The collected smoke was clearly different to the control sample. Stability of the smoke sample was proven by congruent control measurements as late as 24 h after sampling. The further measurements were for logistic reasons performed approximately 5 hours after sampling.

**Figure 3:**
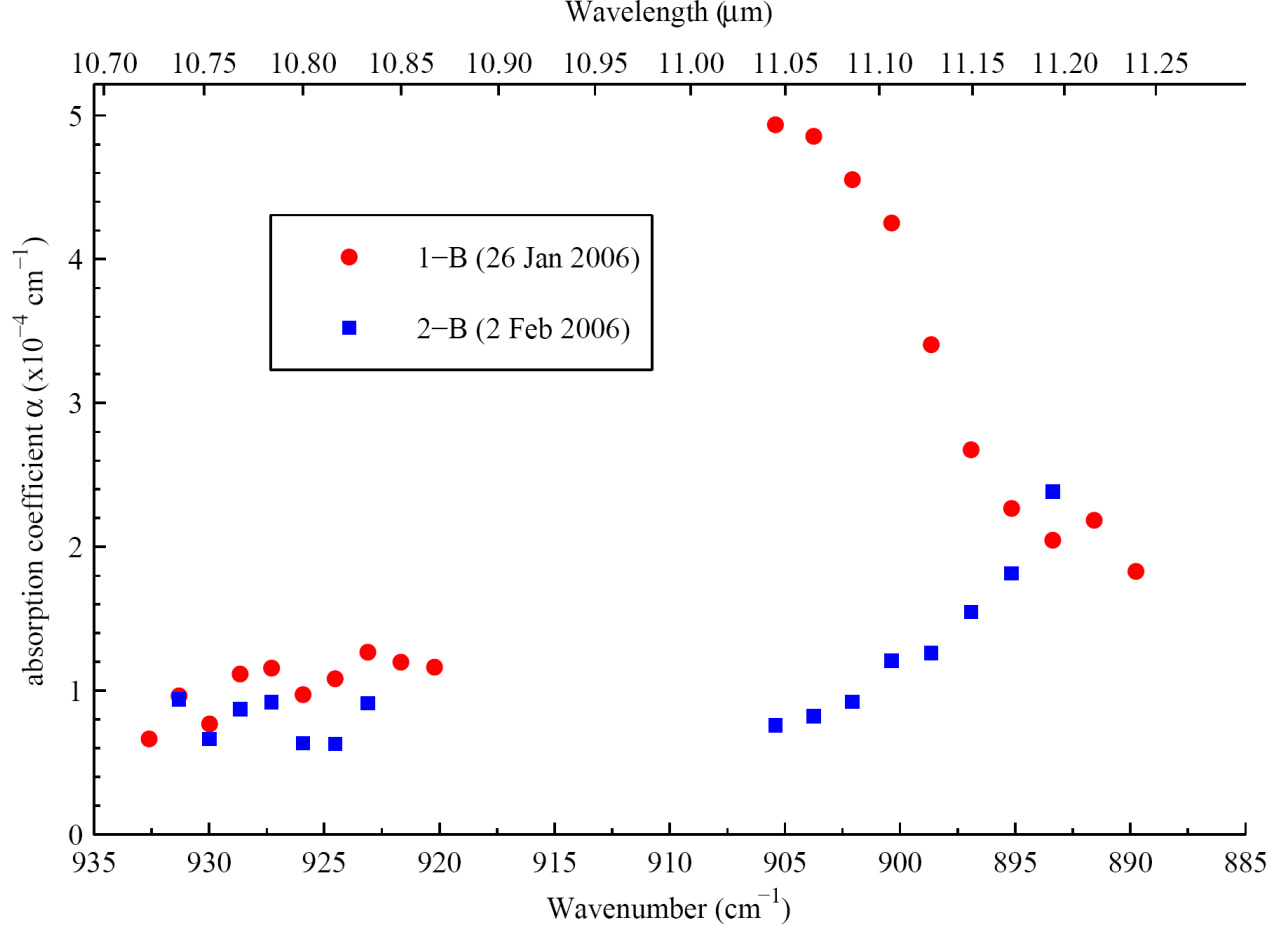
**Photoacoustic absorption spectra of two different patients**. The displayed photoacoustic absorption spectra originate from operations on two different patients (1 and 2). Striking differences were found in the absorption spectra between wavelengths of 890 to 905 cm^-1^.

Fig 3 displays the measurements of 26^th ^of January and 2^nd ^of February. Interestingly, different spectra were detected between the two patients. The photoacoustic spectrometer detected besides a dominating CO_2 _absorption one broad absorption band which was confirmed by FTIR spectral analysis. Apart from 100 ppm H_2_O vapour several other substances were indicated by their respective absorption spectra (Fig 3). Comparing them to the absorption spectra of 32 substances found in previous studies [5,6], neither toxic substances like phenol or indole nor other known substances could unequivocally be identified. The substances therefore are unknown.

## Discussion

We examined the composition of surgical smoke produced during laparoscopic colonic resection using a bipolar vessel sealing device. We detected broad absorption features associated to several molecules. These substances do not correspond to known toxins and have not yet been identified.

We included only four patients in this pilot study. This is in accordance to comparable studies [5,6]. Our measurements were stable and reproducible but failed to answer the original question. Further similar studies at this stage are in our opinion not only unnecessary but also unethical. Ongoing studies of our group are currently trying to answer the open questions with a modified experimental set-up based on the experience of this pilot study [11].

Why did we fail to identify compounds reported by others [5-7]?

First, the smoke composition and quantity depends largely on the combusted tissue and the employed instrument [4,11]. Hensman et al. produced surgical smoke in their *in vitro *study on *porcine *liver, while Hollmann et al. sampled gas emission during open reduction mammoplasty, both using conventional electrocautery [5,6]. Smoke production was likewise heavier in those studies due to the experimental set-up and the type of operation. Furthermore, our gas samples were produced in a CO_2 _atmosphere and are therefore very likely to be different to samples from other studies [4-6]. Several molecules were found by *in vitro *studies of our group under room air [11]. Due to relatively low concentrations in the samples of this study – reflecting however laparoscopic reality – sampling errors can not be excluded. However, we are not aware of any other data on the composition of gas generated by a bipolar vessel sealing device during laparoscopic surgery.

Next, Hensman et al. used gas chromatography-mass spectrometry for analysis [6]. This method requires elaborate sample preparations delivering mainly qualitative results. We used therefore optical techniques that have the advantage of high sensitivity and specifity without need of sample preparation [5,11]. However, spectral range is limited and technical performance is highly dependent on appropriate laser and detection schemes. The optimal detection technique needs still to be defined and might probably depend on the studied problem and the experimental set-up.

## Conclusion

In this study, the use of a vessel sealing device during laparoscopic surgery did not produce known toxic substances in relevant quantity. Ongoing *in vitro *and *in vivo *studies aim to further examine the composition of gas samples produced by different instruments during open and laparoscopic surgery. Meanwhile, intermittent or continuous evacuation of surgical smoke from a cannula with or without add-on filters seems a simple measure to prevent toxic effects, trocar metastases and even to shorten operation time by improving surgeon's vision [4].

## Competing interests

The authors did not receive any financial support from the manufacturers of the surgical instruments.

## Authors' contributions

MH initiated and designed the study and drafted the manuscript. MWS carried out the studies, analyzed the data and helped to draft the manuscript. ND conceived of the study, and participated in its design and coordination and helped to draft the manuscript. MG contributed substantially to acquisition of the data and provided the figures. PAC participated in coordination of the study and helped to draft the manuscript. DH designed the study, carried out the studies and helped to draft the manuscript. All authors read and approved the final manuscript.
